# Identifying the patterns and sizes of the first lumpy skin disease outbreak clusters in Northern Thailand with a high degree of dairy farm aggregation using spatio-temporal models

**DOI:** 10.1371/journal.pone.0291692

**Published:** 2023-11-15

**Authors:** Wittawat Modethed, Tawatchai Singhla, Kittikorn Boonsri, Kidsadagon Pringproa, Nattawooti Sthitmatee, Paramintra Vinitchaikul, Chalutwan Sansamur, Khwanchai Kreausukon, Veerasak Punyapornwithaya

**Affiliations:** 1 Faculty of Veterinary Medicine, Chiang Mai University, Chiang Mai, Thailand; 2 Ruminant Clinic, Department of Food Animal Clinics, Faculty of Veterinary Medicine, Chiang Mai University, Chiang Mai, Thailand; 3 Research Center of Veterinary Biosciences and Veterinary Public Health, Chiang Mai University, Chiang Mai, Thailand; 4 Center of Veterinary Diagnosis and Technology Transfer, Faculty of Veterinary Medicine, Chiang Mai University, Chiang Mai, Thailand; 5 Department of Veterinary Biosciences and Veterinary Public Health, Faculty of Veterinary Medicine, Chiang Mai University, Chiang Mai, Thailand; 6 Laboratory of Veterinary Vaccine and Biological Products, Faculty of Veterinary Medicine, Chiang Mai University, Chiang Mai, Thailand; 7 Akkhararatchakumari Veterinary College, Walailak University, Nakhon Si Thammarat, Thailand; Beni Suef University Faculty of Veterinary Medicine, EGYPT

## Abstract

Lumpy skin disease (LSD) is one of the most important notifiable transboundary diseases affecting cattle in many parts of the world. In Thailand, LSD outbreaks in cattle farming areas have been reported in 69 out of 77 provinces, indicating a serious nationwide situation. Understanding the dynamics of spatial and temporal LSD epidemic patterns can provide important information on disease transmission and control. This study aims to identify spatial and temporal clusters in the first LSD outbreaks in dairy farming areas with a high degree of aggregation in Northern Thailand using spatio-temporal models. The data were obtained from an official LSD outbreak investigation conducted between June and August 2021 on dairy farms (n = 202). The outbreak of LSD was confirmed by employing clinical observations and laboratory analysis. The spatio-temporal models including space-time permutation (STP), Poisson, and Bernoulli were applied to the outbreak data with the settings of 10%, 25%, and 50%, respectively, for the maximum reported cluster size (MRCS). Overall, the number of most likely and secondary clusters varied depending on the model and MRCS settings. All MRCS settings in the STP model detected the most likely clusters in the same area and the Poisson models in different areas, with the largest being defined by a 50% MRCS. Although the sizes of the most likely clusters identified by the Bernoulli models were different, they all had the same cluster period. Based on the sizes of the detected clusters, strict LSD insect-vector control should be undertaken within one kilometer of the outbreak farm in areas where no LSD vaccination has been administered. This study determines the sizes and patterns of LSD outbreak clusters in the dairy farming area with a high degree of farm aggregation. The spatio-temporal study models used in this study, along with multiple adjusted MRCS, provide critical epidemiological information. These models also expand the options for assisting livestock authorities in facilitating effective LSD prevention and control programs. By prioritizing areas for resource allocation, these models can help improve the efficiency of such programs.

## Introduction

Lumpy skin disease (LSD) is an important viral infectious disease in domestic cattle [1], caused by the LSD virus, a member of the *Capripoxvirus* genus of *Poxviridae* [2]. The disease is characterized by raised, circular, firm nodules on the skin and mucosal surface with a diameter of 1 to 5 cm, enlargement of superficial lymph nodes, swelling of the limbs or lower body, lachrymation and nasal discharge, and decreased milk production [3–5]. The morbidity rate ranges from 2 to 45% with mortality being less than 10% [5]. Blood-sucking arthropods such as stable flies, mosquitoes, biting midges [6–8], and ticks [9] play an important role in disease transmission. The LSD causes a significant negative economic impact on cattle production, smallholder farmer livelihoods, and international trade [10]. Importantly, LSD is listed as a notifiable disease by the World Organization for Animal Health [11].

LSD outbreaks have spread from Africa to the Middle East, Europe, and Asia [11]. Several Asian countries experienced LSD outbreaks between 2019 and 2021. In 2019, Bangladesh reported the first Asian outbreak [12]. Later, several LSD outbreaks were reported in China [13], India [14], Nepal [15], Bangladesh [16, 17], Pakistan [18], Vietnam [19], Myanmar [20], Malaysia [21], Hong Kong [22], Laos [23], and Taiwan [24].

In March 2021, the first LSD outbreak was found in cattle farms in the northeastern region of Thailand [25], with subsequent outbreaks being reported in several regions throughout Thailand over a period of more than 11 months [26–29]. Lamphun is considered one of the most important dairy farming regions in Northern Thailand. due to its large milk production capacity [30]. In similarity to other areas in the country, Lamphun is densely populated with dairy farms Since numerous dairy farms were affected by LSD, this area was acknowledged as having one of the most significant outbreaks in Northern Thailand.

The success of disease control strategies depends on a thorough understanding of the epidemiology of the disease. In spatial epidemiology, better knowledge of the spatial and temporal distribution dynamics and the identification of disease clusters can provide crucial information for disease transmission and control [31]. Scan statistics have been increasingly used for the detection and evaluation of disease clusters. These methods are recommended for use as a standard tool in outbreak investigation [32]. Generally, they are utilized to investigate the purely temporal, spatial, or spatio-temporal patterns of disease clusters [33–35]. There are three types of spatio-temporal analyses: space-time permutation (STP), Poisson, and Bernoulli models offered by the SaTScan™ software [36]. A basic understanding of parameter settings, such as the temporal period and spatial scanning window size, is necessary for performing the spatio-temporal analysis [37, 38]. The value of the temporal window size setting is often determined by considering the incubation period of the disease. The spatial scanning window size can be configured by specifying the maximum value of the scanning window size (MSWS). The MSWS is set to use 50% of the total population by default. It can, however, be configured to use different values other than default. Nevertheless, if the setting of the MSWS is made arbitrarily, results can be misleading. For example, if the MSWS is high, the reported cluster is likely to be very large, including non-informative areas. However, if the MSWS value is low, many small clusters are reported, making them less meaningful [39]. Although some studies seem to achieve good results with different MSWS values, repeatedly performing spatial cluster detection analyses by varying the MSWS values leads to a multiple testing problem [40]. Therefore, it is recommended that analyses be performed by varying the values of the maximum reported cluster size (MRCS) rather than the MSWS values [41]. When conducting the analysis, it is important to examine the results obtained from different MRCS settings. This is because the values of the MRCS can have an impact on the cluster size [40–42].

Since there are few publications on LSD in Thailand, knowledge gaps exist on the spatial epidemiology of LSD. To the researchers’ knowledge, only two studies in Thailand have used spatio-temporal approaches to analyze LSD outbreaks: one on beef cattle farms [25] and another on dairy farms in the northeastern region [43]. However, the results from the beef farm region may not be generalizable to the dairy farm region. In addition, the geographical characteristics of dairy farming regions and the degree of aggregation differ between dairy farms in the north and northeast. Thus, it is imperative to conduct an investigation into the epidemiology of LSD outbreaks in the northern region. Furthermore, studies on the use of spatio-temporal analysis with varying MRCS for animal disease data are very limited. Research employing spatio-temporal models with different MRCS settings would be beneficial not only in providing a better understanding of LSD epidemiology, but also information on the size of outbreak clusters and the dispersion of disease outbreaks.

This study aims to identify spatial and temporal clusters of LSD outbreaks in an area where a high degree of farm aggregation exists, using three spatio-temporal models with varying MRCS. The detection of LSD clusters would support livestock authorities in communicating the risk of LSD, establishing effective strategies to control and prevent outbreaks, as well as prioritizing the allocation of resources to high-risk areas.

## Materials and methods

### Study area and case definition

**Fig 1:**
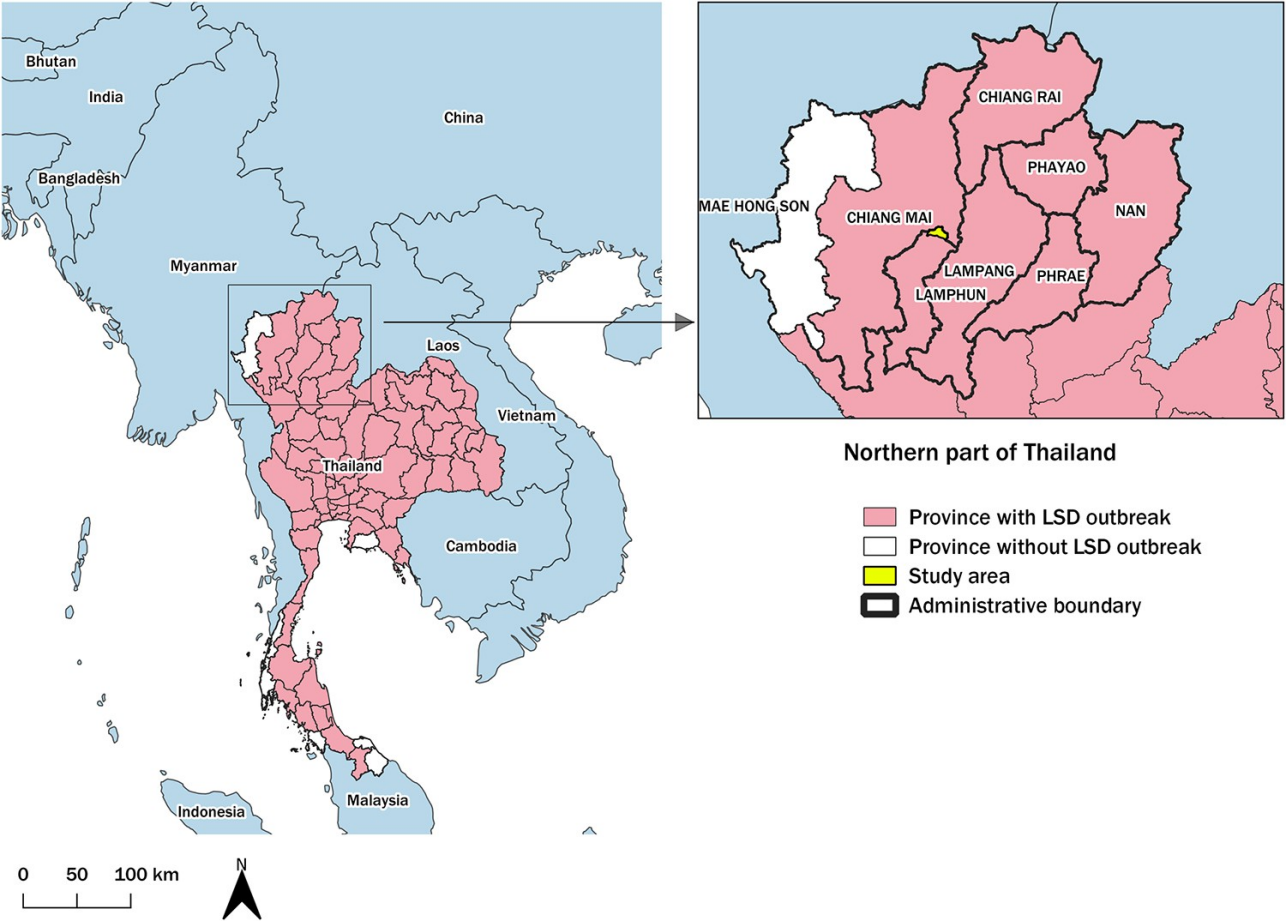
Study area. Provinces in Thailand reporting lumpy skin disease outbreaks (left). The study area is highlighted in yellow. The area with a thick border depicts the northern region of Thailand (right). The map was generated using QGIS (version 3.18.2), QGIS Geographic Information System, and Open–Source Geospatial Foundation Project, and all content is licensed under the Creative Commons Attribution–Share Alike 3.0 license (CC BY–SA), available at (https://qgis.org). In addition, geographical materials used for creating the map (e.g., shape file) were supported by Chiang Mai University. The authors specify that this figure is licensed under CC BY 4.0.

This study was undertaken in Ban Thi District (18.65167 N, 99.12500 E), Lamphun Province, Northern Thailand. This district covers 122.45 Km^2^ bordering San Kamphaeng and Mae On Districts, Chiang Mai Province [29], and is considered to be one of the largest dairy farming areas in Northern Thailand, comprising 202 farms and 10,462 heads of dairy cattle. This area was reported as having the first LSD outbreak in Northern Thailand [29]. Fig 1 depicts Lamphun Province and other provinces where outbreaks of LSD were officially reported by the Department of Livestock Development (DLD). Since the outbreak was the first to occur in this region, the cattle were considered naive as they had never been previously exposed to LSD.

Furthermore, local livestock authorities in this study region conducted control measures based on DLD recommendations [25, 27]. Animal movements were restricted, live cattle markets were closed, pesticides were used to reduce insect vectors, and farmers were encouraged to improve farm biosecurity. It is important to mention that the LSD vaccine was not available for use in this area during the outbreak period. This is because LSD had only been discovered in Thailand a few months before the outbreak occurred in the study area. In response to the outbreak, an investigation was conducted between June and August 2021 based on two different approaches: passive and active. The notification of district livestock officers by dairy farmers was considered a passive approach, whereas the survey conducted by veterinary officers to identify LSD cases in dairy farms represented an active method. Based on this approach, all dairy farms in the district were investigated. The veterinarians conducting the survey were under the authority of the DLD and received special training prior to conducting the LSD outbreak investigation.

Under the outbreak investigation implemented by livestock authorities, an outbreak of LSD was defined by at least one head of dairy cattle in a farm showing LSD-liked clinical signs. The signs include raised, circular, firm nodules varying in diameter from 1 to 5 cm on the skin and/or mucosal surface, enlargement of superficial lymph nodes, and swelling of a limb or lower body [5, 44, 45]. Since the outbreaks were found in naive cattle, clinical signs of LSD are clearly visible, as illustrated in several previous publications conducted in Thailand [25–27]. During the outbreak investigations, the livestock authorities observed all the cattle on each farm for signs of LSD.

It is important to note that DLD authorities collected some samples from LSD–suspected dairy farms in the study area, confirming them to be positive by real–time PCR [46]. Details of the methods carried out in the laboratory are described in a previous study [46].

Following the last LSD outbreak at the dairy farm, the local livestock authority monitored the situation for a duration of six months. There was no occurrence of a new LSD outbreak on the farm. Therefore, the investigation was deemed to have encompassed the entire outbreak period.

### Outbreak data

Data for this study were collected by DLD livestock authorities conducting outbreak investigations in compliance with DLD outbreak response protocols. The data gathered from each farm consisted of the geographical location (latitude and longitude), total number of cattle, number of cattle showing clinical signs of LSD, and number of cattle dying as a result of LSD [29]. It should be noted that no personal details of the farm owner (e.g., name, age, and gender) were included in this study.

### Descriptive data analysis

A graphical representation was generated to visually depict the total number of cattle and the total number of cattle manifesting symptoms of LSD for each individual farm. The epidemiological parameters, including morbidity, mortality, and herd attack rates, were calculated. The morbidity rate was determined by dividing the number of cattle from LSD–affected farms showing clinical signs of the disease by the total number of cattle in those farms [47]. The mortality rate was defined as the number of cattle showing clinical signs of LSD and subsequently dying, divided by the total number of cattle on those farms [47, 48]. Also, the case fatality rate was defined as the number of cattle dying from LSD divided by the number of cattle showing clinical signs of the disease [47, 49]. Moreover, the herd attack rate was calculated by dividing the total number of LSD outbreak farms by the total number of farms in the study area [50]. For calculating morbidity, mortality, and case fatality rates, this study did not utilize data from a specific time point (e.g., date of investigation). Instead, these rates were calculated from data collected over the period of the outbreak. This approach was adopted due to the ongoing monitoring efforts by local veterinary authorities and dairy farmers to identify new cases of LSD on the farms. Similarly, the attack rate of the herd was determined by considering the duration of the outbreak. Furthermore, the epidemic curve was created to depict the number of LSD outbreak farms, providing a visual representation of an epidemiological feature of LSD outbreaks at the farm level.

### Spatio-temporal analysis and parameter setting

The space-time scan statistics available in the SaTScan™ software [33, 51], including space-time permutation (STP), Poisson, and Bernoulli models, were used for the detection and evaluation of the disease clusters [36]. Furthermore, different MRCS values were applied, namely 50%, 25%, and 10% [41, 52].

The STP model uses only LSD case data with the geographical coordinates and onset date of the outbreaks to determine the outbreak cluster of outbreaks [33]. Whereas the Poisson model compares the number of LSD cases and number of the population at risk within and outside the candidate windows of space and time using the number of LSD cases, total number of cattle at each farm, coordinates of the farm, and the onset date of the outbreak. The Bernoulli model requires data such as the number of LSD cases from LSD outbreak farms, total number of dairy cattle at LSD outbreak and non-LSD outbreak farms, geographical coordinates of the farms, and onset date of the outbreaks to determine the distribution of LSD cases at the location compared to the control [51, 53].

For each spatio-temporal model, the most likely and secondary clusters were defined by the value of the log-likelihood ratio [36]. In STP and Poisson models, under the Poisson assumption, the likelihood function for a specific window is proportional to [36]:

$${\left(\frac{c}{E[c]}\right)}^{c}{\left(\frac{C-c}{C-E[c]}\right)}^{C-c}I()$$ (1)

where *C* is the total number of LSD cases, *c* refers to the observed number of LSD cases within the space-time window, and *E*[*c*] denotes the covariate-adjusted expected number of LSD cases within the window under the null hypothesis, while *C*−*E*[*c*] is the expected number of cases outside the window. The term *I*() is an indicator function, if the purpose is to scan only for clusters with a high rate, the *I*() is set to 1.

For the Bernoulli model, the likelihood function is written as follows [36]:

$${\left(\frac{c}{n}\right)}^{c}{\left(\frac{n-c}{n}\right)}^{n-c}{\left(\frac{C-c}{N-n}\right)}^{C-c}{\left(\frac{\left(N-n)-(C-c\right)}{N-n}\right)}^{\left(N-n)-(C-c\right)}I()$$ (2)

where *C* and *c* are the total number of LSD cases and the observed number of LSD cases within the window, respectively, while *n* is the total number of cases and controls within the window. The term *N* denotes the combined total number of cases and controls in the data set and *I*() is an indicator function.

The Monte Carlo simulation (number of replications = 999) is used to determine the statistical significance of a cluster being formed by chance [36]. The level of statistical significance was set as α = 0.05. Furthermore, statistically significant secondary clusters are determined and ranked based on their log-likelihood ratio (LLR) values [54].

For all models, the spatial dimension for scanning the cluster was kept as 50% of the population at risk, while the MRCS values were set as 50%, 25%, and 10% [41, 52]. In addition, the temporal dimension was set as 50% of the study period, and the temporal unit as seven days based on the LSD incubation period [55]. Other analytic options were left at their default values. Technically, the MRCS setting is an option under the advanced output feature window of the SaTScan™. The option to “Report only clusters smaller than” was selected. For each model and round, 10%, 25%, and 50% of the population at risk were used as inputs. Furthermore, clusters defined by the spatio-temporal models were mapped using the Quantum Geographic Information System (QGIS), an open-source software. The shape files for administrative divisions used by the QGIS were obtained from Chiang Mai University.

## Ethics statement

The study was reviewed and approved by the Institutional Review Board, Faculty of Veterinary Medicine, Chiang Mai University under, reference number HS1/2565. This study utilized the secondary outbreak investigation data collected by veterinary authorities, which included geographical data, farm identification numbers, and the number of cattle involved in outbreaks of LSD. The authors had no role in the investigation of the outbreak and were not involved with the animals. In addition, no demographic information regarding farm owners was utilized in this study.

## Results

### Epidemiological features of LSD outbreaks

**Fig 2:**
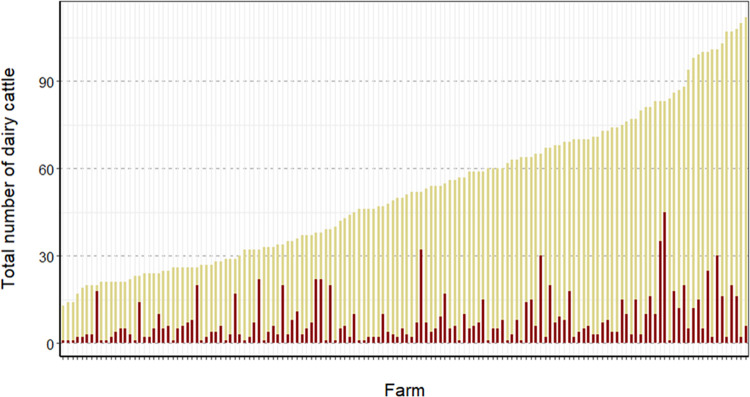
Number of cattle with and without clinical signs of lumpy skin disease (LSD). For each farm, the red bar represents the total number of cattle with clinical signs of LSD, while the yellow bar depicts the number of cattle without clinical signs of LSD.

The first LSD outbreak in a dairy farming area in the northern region was reported on June 6, 2021, in Ban Thai District, Lamphun Province. Data from June 6 to August 27, 2021, showed that 145 farms out of 202 were affected by LSD, which means that the herd attack rate was 72%. Fig 2 illustrates the total number of dairy cattle without clinical signs and those showing clinical signs of LSD at each dairy farm. Overall, the morbidity, mortality, and case fatality rates were 11.30% (n = 1,183/10,462), 0.34% (n = 36/10,462), and 3.04% (n = 36/1,183), respectively. The highest and lowest morbidity rates were 90 and 1.19, respectively.

**Fig 3:**
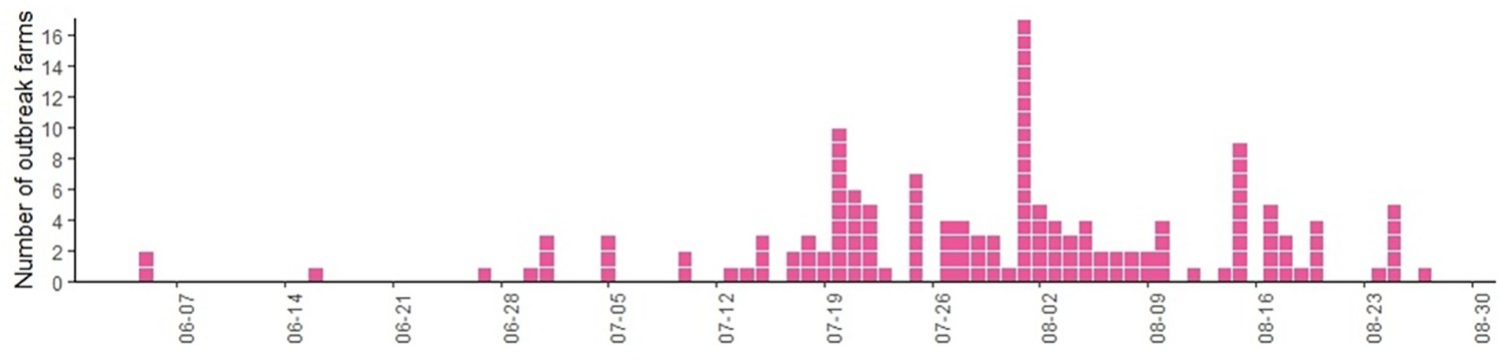
The epidemic curve. The epidemic curve shows the number of dairy farms affected by lumpy skin disease (LSD) during the first LSD outbreak in the dairy farming areas of Northern Thailand, in 2021. This curve depicts the temporal epidemiological characteristics observed throughout the entirety of the LSD outbreak period, covering June to August 2021.

According to the epidemic curve, the number of LSD outbreak farms started to increase in late June, rising significantly during the month of July, and peaking in early August. In late August, there was a decline in the number of outbreak farms (Fig 3).

The farms in the outbreak area were located in close proximity. The mean and standard deviation of the distance from a particular farm to the nearest neighboring farms were 0.13 ± 0.26 km, ranging from 0.01 to 3 km. The majority of dairy farms were located in the vicinity of the district border of San Kamphaeng, Chiang Mai Province.

### Spatio-temporal clusters

In the spatio-temporal analysis, only one cluster was determined to be the most likely cluster, while the number of secondary clusters varied depending on the model used and its settings [36].

**Fig 4:**
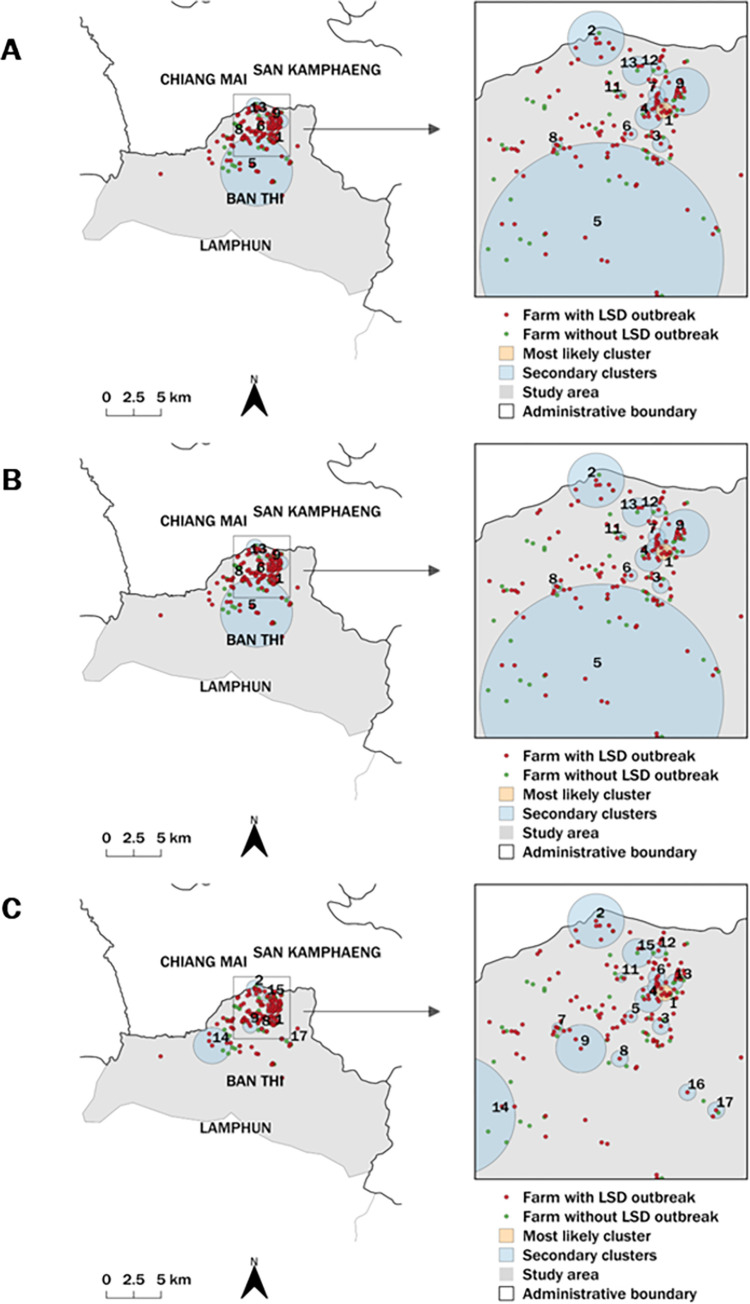
Spatio–temporal maps of the STP model. The most likely cluster and secondary clusters detected by the STP model with the MRCS specified at 50% (A), 25% (B), and 10% (C). The map was generated using QGIS (version 3.18.2), QGIS Geographic Information System, and Open–Source Geospatial Foundation Project, and all content is licensed under the Creative Commons Attribution–Share Alike 3.0 license (CC BY–SA), available at (https://qgis.org). In addition, geographical materials used for creating the map (e.g., shape file) were supported by Chiang Mai University. The authors specify that this figure is licensed under CC BY 4.0.

**Fig 5:**
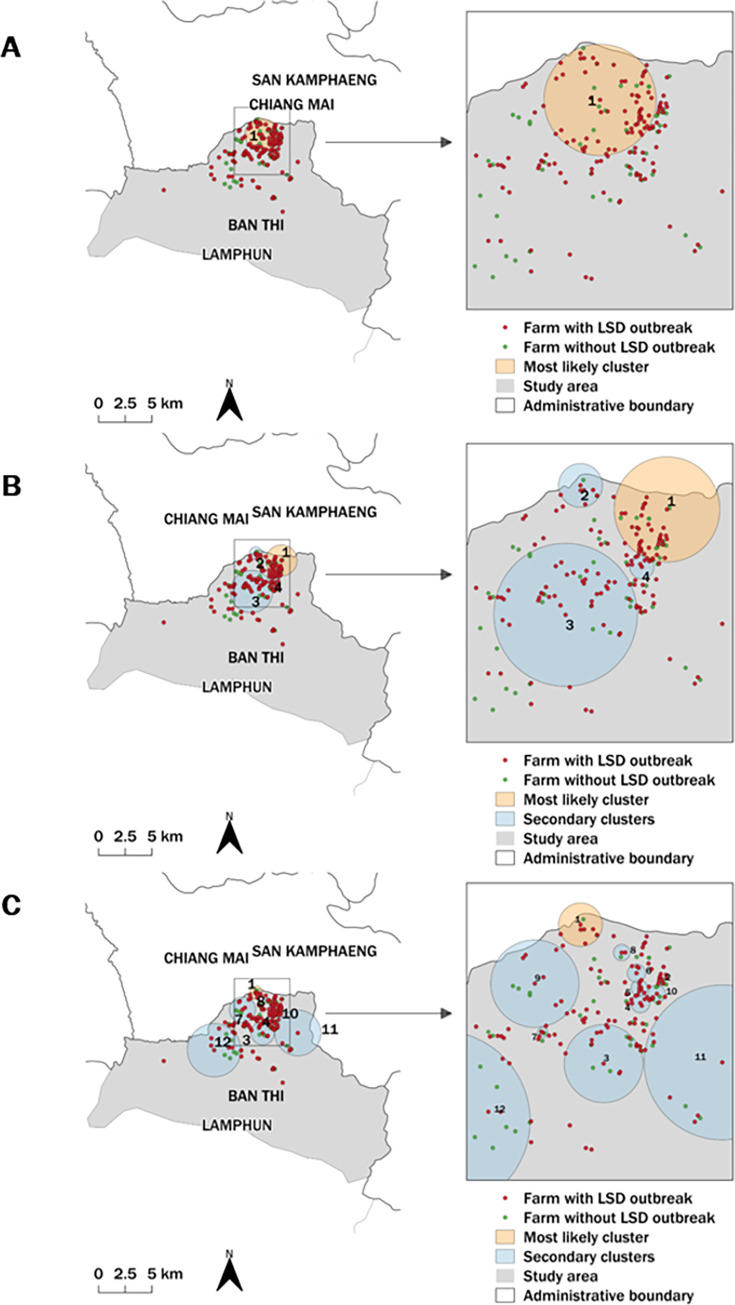
Spatio–temporal maps of the poisson model. The most likely cluster and secondary clusters detected by the Poisson model with the MRCS specified at 50% (A), 25% (B), and 10% (C). The map was generated using QGIS (version 3.18.2), QGIS Geographic Information System, and Open–Source Geospatial Foundation Project, and all content is licensed under the Creative Commons Attribution–Share Alike 3.0 license (CC BY–SA), available at (https://qgis.org). In addition, geographical materials used for creating the map (e.g., shape file) were supported by Chiang Mai University. The authors specify that this figure is licensed under CC BY 4.0.

**Fig 6:**
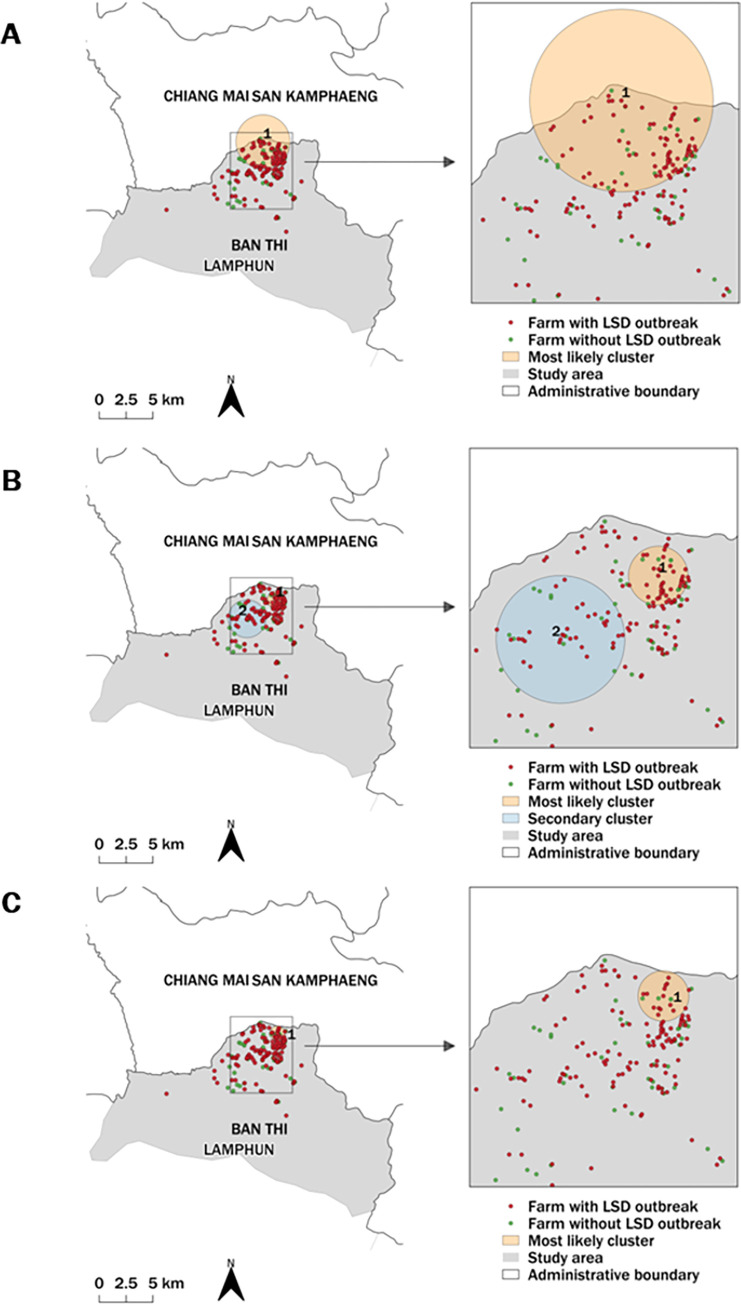
Spatio–temporal maps of the Bernoulli model. The most likely cluster and secondary clusters detected by the Bernoulli model with the MRCS specified at 50% (A), 25% (B), and 10% (C). The map was generated using QGIS (version 3.18.2), QGIS Geographic Information System, and Open–Source Geospatial Foundation Project, and all content is licensed under the Creative Commons Attribution–Share Alike 3.0 license (CC BY–SA), available at (https://qgis.org). In addition, geographical materials used for creating the map (e.g., shape file) were supported by Chiang Mai University. The authors specify that this figure is licensed under CC BY 4.0.

The STP model identified the most likely and 12 secondary clusters based on the setting of 50% and 25% MRCS whereas the 10% MRCS yielded 16 clusters (Fig 4). The Poisson model identified the three most likely and 11 secondary clusters with 25% and 10% MRCS, respectively, while no secondary cluster was found with 50% MRCS (Fig 5). The Bernoulli model provided only one secondary cluster from the setting of 25% MRCS (Fig 6).

Regarding the STP model results, all MRCS settings yielded similar cluster areas in the same period (Table 1). Contrarily, different MRCS setting values for the Poisson model resulted in the most likely clusters being in different locations, with the largest most likely cluster resulting from the 50% MRCS configuration (Table 2). The primary clusters detected by the Poisson model were larger and included more LSD cases and LSD outbreak farms than those detected by the STP model. All MRCS setting values of the Bernoulli model identified the most likely clusters in the same period but the sizes varied (Table 3).

**Table 1:** The most likely clusters detected by the space–time permutation scan statistic model for LSD outbreaks at dairy farms in Northern Thailand, 2021.

MRCS ^1^	Cluster time	Centroid (X, Y) / Radius (km)	Number of farms	O ^2^	E ^3^	O/E ratio ^4^	Test Statistics	p-value
50%	1–8 Jun 2021	18.695466 N, 99.166620 E/ less than 0.1 km	1	35	1.61	21.75	74.90	<0.01
25%	1–8 Jun 2021	18.695466 N, 99.166620 E/ less than 0.1 km	1	35	1.61	21.75	74.90	<0.01
10%	1–8 Jun 2021	99.166620 E/ less than 0.1 km	1	35	1.61	21.75	74.90	<0.01

^1^ MRCS = maximum reported cluster size

^2^ O = observed case

^3^ E = expected case

^4^ O/E ratio = the ratio of observed cases/expected cases.

**Table 2:** The most likely clusters detected by the Poisson model for LSD outbreaks at dairy farms in Northern Thailand, 2021.

MRCS ^1^	Cluster time	Centroid (X, Y)/ Radius (km)	Number of farms	O ^2^	E ^3^	O/E ratio ^4^	RR ^5^	LLR ^6^	p-value
50%	14–20 Jul 2021	18.699923 N, 99.158230 E/ 1.05 km	58	159	60.21	2.64	2.91	60.32	< 0.001
25%	28 Jul–3 Aug 2021	18.703724 N, 99.170127 E/ 0.99 km	37	162	65.40	2.48	2.72	54.87	< 0.001
10%	14–20 Jul 2021	18.707794 N, 99.154751 E/ 0.41 km	7	80	20.50	3.90	4.12	51.05	<0.001

^1^ MRCS = maximum reported cluster size

^2^ O = observed case

^3^ E = expected case

^4^ O/E ratio = the ratio of observed cases/expected cases

^5^ RR = relative risk

^6^ LLR = log–likelihood ratio.

**Table 3:** The most likely clusters detected by the Bernoulli model for LSD outbreaks at dairy farms in Northern Thailand, 2021.

MRCS ^1^	Cluster time	Centroid (X, Y) / Radius (km)	Number of farms	O ^2^	E ^3^	O/E ratio ^4^	RR ^5^	LLR ^6^	p-value
50%	28 Jul–3 Aug 2021	18.707028 N, 99.157001 E/ 1.71 km	90	21	15.56	1.35	1.41	6.75	0.03
25%	28 Jul–3 Aug 2021	18.699550 N, 99.164704 E/ 0.55 km	45	14	10.37	1.35	1.39	4.39	0.29
10%	28 Jul–3 Aug 2021	18.702710 N, 99.165604 E/ 0.47 km	18	8	5.93	1.35	1.37	2.46	0.96

^1^ MRCS = maximum reported cluster size

^2^ O = observed case

^3^ E = expected case

^4^ O/E ratio = the ratio of observed cases/expected cases

^5^ RR = relative risk

^6^ LLR = log–likelihood ratio.

It is highlighted that the most likely clusters from all MRCS settings for the STP, Poisson, and Bernoulli spatio-temporal models had radii of less than 1.72 km, with the majority having radii of less than 1 km.

Based on the results for all models (S1–S3 Tables), the secondary clusters had radii ranging from 0.10–2.23 km. The STP model produced more secondary clusters than the other models. It should be noted that the STP models produced the same results for 50% and 25% MRCS, whereas the 10% MRCS produced more significant secondary clusters than the other two settings. Most of the secondary clusters detected by the SPT model had radii of less than 0.5 km. Overall, the Poisson model identified more secondary clusters with a larger radius compared to the STP model. The Bernoulli method detected a significant secondary cluster when a 50% MRCS was set. However, none of the other MRCS settings utilized in this method provide any significant clusters.

## Discussion

This study utilized spatio-temporal models with varying MRCS settings to identify spatio-temporal clusters of LSD outbreaks at highly aggregated dairy farms in the northern region of Thailand.

Due to the availability of data from both LSD outbreak and non-LSD outbreak farms, all three important spatio-temporal models offered by the SaTScan™ were able to be utilized [36]. Rather than using one fixed value to obtain disease clusters, different MRCS settings [41, 52, 56] were applied to generate the various results. This approach is based on the presumption that it is difficult to determine the most appropriate pattern based on a single model and single choice of the spatio-temporal model, and thus one should conduct various spatio-temporal analyses by changing the shape, size, and density of event distribution [37, 57]. In this study, according to different model parameter settings, LSD outbreak clusters with different sizes were identified.

The results of all models indicated that, as expected, a smaller MRCS setting would be more likely to result in a smaller cluster size. Also, the number of clusters varied depending on the model and MRCS setting. The findings of this study demonstrated that the MRCS settings defined in the STP model produced identical primary clusters; however, each setting provided a different set of secondary clusters. Unlike the STP, each MRCS parameter setting for the Poisson and Bernoulli models identified a different set of the most likely clusters. Furthermore, it was demonstrated that the sizes of the clusters defined by the Bernoulli model varied, but all had the same cluster period. Based on the findings of this study, the STP model offered an advantage since it could detect the most likely spatio-temporal cluster earlier than other models. This finding was concordant with the previous study, indicating that the STP model may be an effective tool for the early detection of disease outbreaks [33]. Nevertheless, the most likely clusters detected by the STP model contained dairy cattle from only one farm. As a result, this finding should be interpreted cautiously because these clusters may be too small. The approach by which STP scanning windows look for the area with the highest number of cases across the entire region without considering the population at risk may lead to the detection of small clusters. Therefore, the STP scanning windows will identify the region with the highest number of cases, despite them coming from a single farm. This circumstance, however, was not observed in the Poisson model for this outbreak data since the clusters defined by the model had larger radii and contained more farms. Notably, the STP identified smaller clusters than the Poisson model and may not be generalizable to other outbreak data, since these models generally perform differently depending on the data. Furthermore, while the sizes of the clusters identified by the Bernoulli model varied depending on the MRCS setting values, they all had the same cluster period, encompassing the peak of the epidemic curve. The Bernoulli model has an advantage over the STP and Poisson models because it includes both outbreak and non-outbreak farms in its results, allowing for additional investigation, such as a case-control study to investigate the risk factors associated with LSD outbreaks in the study area [58, 59]. Secondary clusters are also worth investigating because some clusters may be relevant to the objectives of the decision-makers. For instance, if the authorities focus primarily on high-risk areas, they might visit all farms in secondary clusters rather than all farms in the outbreak area to conduct further investigations or engage in other activities, such as organizing focus groups for LSD risk communication and demonstrating how to control LSD insect vectors.

It has been suggested that very small clusters may not be informative, and very large clusters not meaningful [39]; thus, it is recommended that different parameter settings be used to produce better results [41]. Given that each nation has a different policy and level of budget and resources, such an approach gives decision-makers a chance to choose the option most relevant to the achievement of their goals. For instance, the most relevant clusters can be chosen for the implementation of disease control strategies, such as strictly controlling animal movement, controlling vectors within a reference radius, or vaccinating rings around the area.

It is acknowledged that insect vectors play an important role in the short-distance spread of LSD, whereas the movement of infected animals is associated with long-distance spread [9, 60, 61]. A previous study determined the transmission of LSD via insects by estimating the reproduction numbers (R_0_) of some important insect vectors, such as *Stomoxys calcitrans* (stable fly) and *Culicoides nubeculosus* (biting midge) [62]. The estimated R_0_ values from that study were greater than 1, indicating the significant transmission ability of those insects [61, 62]. Moreover, the transmission from infected animals to other animals has been addressed in several studies [63]. A recent study indicated that the transmission of LSD via *Stomoxys calcitrans* occurred from the experimentally infected animals to naive cattle [64]. Indeed, although it is generally acknowledged that vaccination is considered the most effective preventative measure and vector control should be viewed as a supportive measure [9], insect control offers the means to prevent the spread of disease from infected animals to other animals in an area with no LSD outbreak or where animals in the area have not been immunized through vaccination. The extensive spread of LSD among cattle at outbreak farms in Thailand is consistent with this scenario [28]. Thus, in response to the country’s first outbreak in which no LSD vaccination was used, Thailand’s livestock authority implemented insect-vector control as a control measure, in addition to restrictions on animal movement, the closure of live cattle markets, and the use of disinfectant [46]. One of the recommendations is to restrict the movement of animals within a 50-kilometer radius of the outbreak farm. Nevertheless, unlike animal movement control, the authorities make no recommendations regarding insect control radius sizes, despite their emphasis on insect-vector control. This might be because insufficient information exists to support the decision. The results of this study, focusing on spatio-temporal clusters, may offer some guidance regarding the level of insect control.

In the study area, animal movement among farms is restricted, and the average distance between farms is relatively short. Thus, the chance of LSD spreading through animal movement is less likely than through insect vectors. In the circumstances, it could be speculated that the close proximity of dairy farms is a potential factor associated with LSD outbreaks. This condition could facilitate the transmission of LSD via insect vectors if these vectors are inadequately controlled or not controlled at all. In addition, the findings of this study show that the vast majority of the most likely clusters identified by all models have radii of less than 1 km. Therefore, in a region with a high concentration of farms where animal movement is restricted, a farm in Thailand with non-LSD-vaccinated animals located within approximately 1 km of the outbreak farm may be advised to implement stringent insect-vector control to reduce the likelihood of LSD spreading.

Notably, during the outbreak under study, the herd attack rate was greater than 70%, implying high disease transmutability in this area. Furthermore, the morbidity and mortality rates were in the range of those reported in previous studies, indicating that morbidity was 2–45% and mortality less than 10% [55, 65]. However, the mortality and morbidity rates were lower than those reported in the LSD outbreak study conducted in the dairy farming area in the northeastern region of Thailand [43]. This could be attributed to variations in various factors such as geographical characteristics, insect vector abundance, and farm management practices. Although the use of spatio-temporal models has been demonstrated in a previous study conducted in Thailand [43], two major points need to be acknowledged with this study. First, the average distance observed in this study between one farm and the neighboring farm was approximately eight times shorter than that observed in the previous study, implying a higher degree of farm aggregation. Second, unlike the previous study, in which basic settings are employed to detect spatio-temporal clusters, the methodology used in this study is based on advanced settings. Thus, the findings obtained from the present study expand the existing knowledge on spatio-temporal patterns in LSD outbreaks.

This study has some limitations. The majority of LSD cattle cases were diagnosed based on clinical signs of LSD. Nonetheless, some cattle from various farms in this area were confirmed as LSD cases after DLD veterinary authorities tested blood and tissue samples from them in the laboratory. Later, this LSD outbreak was officially announced and made public via the DLD website based on the laboratory results previously described [29]. Notably, this limitation is also commonly found in other studies since not all animals or a large percentage of animals in relation to the population are at risk in outbreak areas confirmed as having the disease through laboratory testing [27, 66–69]. Furthermore, the outbreak under study was the first in the area, and therefore most farms in the region were LSD outbreak farms. Due to a low number of control farms (non-LSD outbreak farms) compared to LSD outbreak farms, a case-control study to identify the risk factors associated with outbreaks from the clusters detected by the Bernoulli model cannot be conducted. However, follow-up studies with a sufficient sample size to determine the risk factors for LSD outbreaks in other areas of Thailand are warranted.

## Conclusions

Detecting spatio-temporal disease clusters is essential for comprehending disease patterns and distributions in both space and time. This is the first study to identify spatio-temporal clusters of LSD outbreaks in Northern Thailand with a high degree of dairy farm aggregation. The radii of the most significant disease clusters identified by STP, Poisson, and Bernoulli are less than 0.5 km, implying that close proximity may be associated with LSD transmission, particularly from one farm to another via insect vectors. Based on the size of the spatio-temporal clusters identified in this study, it is suggested that strict insect-vector control be implemented within a one-kilometer radius of an LSD outbreak farm in an area with a high concentration of farms with cattle that have not been previously exposed to the disease. This study provides useful information on the spatial epidemiology of LSD, which is critical for decision-makers or livestock authorities in strengthening disease control and prevention programs.

## Supporting information

S1 Table The most likely clusters detected by the space-time permutation scan statistic model (STP) of the first LSD outbreak in a dairy farming area in Northern Thailand in 2021. (PDF)

S2 Table The most likely clusters detected by the discrete Poisson space-time scan statistic model (Poisson ST) of the first LSD outbreak in a dairy farming in Northern Thailand in 2021. (PDF)

S3 Table The most likely clusters detected by the Bernoulli scan statistic model of the first LSD outbreak in a dairy farming area in Northern Thailand dairy in 2021. (PDF)
