# Supplementary material for: Identifying the patterns and sizes of the first lumpy skin disease outbreak clusters in Northern Thailand with a high degree of dairy farm aggregation using spatio-temporal models
Source: PLoS One. 2023 Nov 15;18(11):e0291692. doi: 10.1371/journal.pone.0291692 (PMC10651038; doi:10.1371/journal.pone.0291692)
Supplement: S1 Table — (PDF) [file pone.0291692.s001.pdf]

Supporting information 1

S1 Table. The most likely clusters detected by space-time permutation scan statistic model (STP) of the first LSD outbreak in Northern Thailand's dairy, 2021.

MRCS[1]	Cluster type	Cluster time	Centroid(X,Y)/ Radius(km)	O[2]	E[3]	O/E ratio[4]	LLR[5]	p-value
**50%**	Most likely cluster	2021/6/1 to 2021/6/8	18.695466 N, 99.166620 E / less than 0.1 km	35	1.61	21.75	74.9	<0.001
	Secondary cluster 2	2021/7/14 to 2021/7/20	(18.707794 N, 99.154751 E) / 0.52 km	82	16	5.12	70	<0.001
	Secondary cluster 3	2021/7/7 to 2021/7/13	(18.689761 N, 99.166006 E) / less than 0.1 km	25	0.8	31.42	62.24	<0.001
	Secondary cluster 4	2021/7/14 to 2021/7/20	(18.694410 N, 99.163907 E) / 0.25 km	69	13.6	5.07	58.04	<0.001
	Secondary cluster 5	2021/8/4 to 2021/8/10	(18.669861 N, 99.155682 E) / 2.23 km	102	29.38	3.47	56.78	<0.001
	Secondary cluster 6	2021/6/30 to 2021/7/6	(18.691439 N, 99.160835 E) / 0.11 km	29	1.67	17.4	55.84	<0.001
	Secondary cluster 7	2021/7/21 to 2021/7/27	(18.698118 N, 99.165304 E) / less than 0.1 km	45	6.17	7.3	51.28	<0.001
	Secondary cluster 8	2021/8/11 to 2021/8/17	(18.689495 N, 99.148023 E) / 0.097 km	35	3.7	9.47	47.82	<0.001
	Secondary cluster 9	2021/7/28 to 2021/8/3	(18.698719 N, 99.170105 E) / 0.45 km	100	34	2.94	43.9	<0.001
	Secondary cluster 10	2021/8/18 to 2021/8/24	(18.696493 N, 99.165203 E) / less than 0.1 km	20	0.94	21.34	42.31	<0.001
	Secondary cluster 11	2021/8/25 to 2021/8/31	(18.698112 N, 99.159180 E) / 0.088 km	15	0.34	43.5	42.03	<0.001
	Secondary cluster 12	2021/8/11 to 2021/8/17	(18.702710 N, 99.165604 E) / 0.14 km	23	2.24	10.28	33.03	<0.001
	Secondary cluster 13	2021/7/28 to 2021/8/3	(18.702290 N, 99.161924 E) / 0.27 km	32	8.12	3.94	20.26	<0.001
**25%**	Most likely cluster	2021/6/1 to 2021/6/8	18.695466 N, 99.166620 E / less than 0.1 km	35	1.61	21.75	74.9	<0.001
	Secondary cluster 2	2021/7/14 to 2021/7/20	(18.707794 N, 99.154751 E) / 0.52 km	82	16	5.12	69.97	<0.001

	Secondary cluster 3	2021/7/7 to 2021/7/13	(18.689761 N, 99.166006 E) / less than 0.1 km	25	0.8	31.42	62.24	<0.001
	Secondary cluster 4	2021/7/14 to 2021/7/20	(18.694410 N, 99.163907 E) / 0.25 km	69	13.6	5.07	58.04	<0.001
	Secondary cluster 5	2021/8/4 to 2021/8/10	(18.669861 N, 99.155682 E) / 2.23 km	102	29.38	3.47	56.78	<0.001
	Secondary cluster 6	2021/6/30 to 2021/7/6	(18.691439 N, 99.160835 E) / 0.11 km	29	1.67	17.4	55.84	<0.001
	Secondary cluster 7	2021/7/21 to 2021/7/27	(18.698118 N, 99.165304 E) / less than 0.1 km	45	6.17	7.3	51.28	<0.001
	Secondary cluster 8	2021/8/11 to 2021/8/17	(18.689495 N, 99.148023 E) / 0.097 km	35	3.7	9.47	47.82	<0.001
	Secondary cluster 9	2021/7/28 to 2021/8/3	(18.698719 N, 99.170105 E) / 0.45 km	100	34	2.94	43.9	<0.001
	Secondary cluster 10	2021/8/18 to 2021/8/24	(18.696493 N, 99.165203 E) / less than 0.1 km	20	0.94	21.34	42.31	<0.001
	Secondary cluster 11	2021/8/25 to 2021/8/31	(18.698112 N, 99.159180 E) / 0.088 km	15	0.34	43.5	42.03	<0.001
	Secondary cluster 12	2021/8/11 to 2021/8/17	(18.702710 N, 99.165604 E) / 0.14 km	23	2.24	10.28	33.03	<0.001
	Secondary cluster 13	2021/7/28 to 2021/8/3	(18.702290 N, 99.161924 E) / 0.27 km	32	8.12	3.94	20.26	<0.001
**10%**	Most likely cluster	2021/6/1 to 2021/6/8	(18.695466 N, 99.166620 E) / less than 0.1 km	35	1.61	21.75	74.89	<0.001
	Secondary cluster 2	2021/7/14 to 2021/7/20	(18.707794 N, 99.154751 E) / 0.52 km	82	16	5.12	69.98	<0.001
	Secondary cluster 3	2021/7/7 to 2021/7/13	(18.689761 N, 99.166006 E) / less than 0.1 km	25	0.8	31.42	62.24	<0.001
	Secondary cluster 4	2021/7/14 to 2021/7/20	(18.694410 N, 99.163907 E) / 0.25 km	69	13.6	5.07	58.04	<0.001
	Secondary cluster 5	2021/6/30 to 2021/7/6	(18.691439 N, 99.160835 E) / 0.11 km	29	1.67	17.4	55.84	<0.001
	Secondary cluster 6	2021/7/21 to 2021/7/27	(18.698118 N, 99.165304 E) / less than 0.1 km	45	6.17	7.3	51.28	<0.001

Secondary cluster 7	2021/8/11 to 2021/8/17	(18.689495 N, 99.148023 E) / 0.097 km	35	3.7	9.47	47.82	<0.001
Secondary cluster 8	2021/6/16 to 2021/6/22	(18.684202 N, 99.158836 E) / less than 0.1 km	14	0.17	80.79	47.74	<0.001
Secondary cluster 9	2021/8/4 to 2021/8/10	(18.685928 N, 99.152085 E) / 0.46 km	42	6.59	6.38	42.96	<0.001
Secondary cluster 10	2021/8/18 to 2021/8/24	(18.696493 N, 99.165203 E) / less than 0.1 km	20	0.94	21.34	42.31	<0.001
Secondary cluster 11	2021/8/25 to 2021/8/31	(18.698112 N, 99.159180 E) / 0.088 km	15	0.34	43.5	42.03	<0.001
Secondary cluster 12	2021/8/11 to 2021/8/17	(18.702710 N, 99.165604 E) / 0.14 km.	23	2.24	10.28	33.03	<0.001
Secondary cluster 13	2021/7/28 to 2021/8/3	(18.697331 N, 99.168418 E) / 0.15 km	55	14.97	3.67	32.26	<0.001
Secondary cluster 14	2021/8/11 to 2021/8/17	(18.674529 N, 99.129612 E) / 1.16 km	23	2.82	8.15	28.27	<0.001
Secondary cluster 15	2021/7/28 to 2021/8/3	(18.702290 N, 99.161924 E) / 0.27 km	32	8.12	3.94	20.26	<0.001
Secondary cluster 16	2021/8/4 to 2021/8/10	(18.678436 N, 99.170618 E) / less than 0.1 km	16	2.11	7.59	18.62	<0.001
Secondary cluster 17	2021/7/28 to 2021/8/3	(18.675360 N, 99.175603 E) / less than 0.1 km	16	4.06	3.94	10.07	0.0024

[1] MRCS=maximum reported cluster size; [2] O=observed case; [3] E=expected case; [4] O/E ratio=the ratio of observed cases/expected cases; [5] LLR=log-likelihood ratio.
