# Supplementary material for: Identifying the patterns and sizes of the first lumpy skin disease outbreak clusters in Northern Thailand with a high degree of dairy farm aggregation using spatio-temporal models
Source: PLoS One. 2023 Nov 15;18(11):e0291692. doi: 10.1371/journal.pone.0291692 (PMC10651038; doi:10.1371/journal.pone.0291692)
Supplement: S2 Table — (PDF) [file pone.0291692.s002.pdf]

Supporting information 2

S2 Table. The most likely clusters detected by discrete Poisson space-time scan statistic model (Poisson ST) of the first LSD outbreak in dairy farming in northern Thailand's 2021

MRCS[1]	Cluster type	Cluster time	Centroid(X,Y)/ Radius(km)	O[2]	E[3]	O/E ratio[4]	RR[5]	LLR[6]	p-value
**50%**	Most likely cluster	2021/7/14 to 2021/7/20	18.699923 N, 99.158230 E / 1.05 km	159	60.21	2.64	2.91	60.32	<0.001
**25%**	Most likely cluster	2021/7/28 to 2021/8/3	18.703724 N, 99.170127 E / 0.99 km	162	65.4	2.48	2.72	54.87	<0.001
	Secondary cluster 2	2021/7/14 to 2021/7/20	18.707794 N, 99.154751 E / 0.41 km	80	20.5	3.9	4.12	51.05	<0.001
	Secondary cluster 3	2021/8/4 to 2021/8/10	18.685928 N, 99.152085 E / 1.34 km	80	24.74	3.23	3.4	40.03	<0.001
	Secondary cluster 4	2021/7/14 to 2021/7/20	18.694021 N, 99.165646 E / 0.23 km	69	23.62	2.92	3.05	29.54	<0.001
**10%**	Most likely cluster	2021/7/14 to 2021/7/20	18.707794 N, 99.154751 E / 0.41 km	80	20.5	3.9	4.12	51.05	<0.001
	Secondary cluster 2	2021/7/28 to 2021/8/3	18.698431 N, 99.169228 E / 0.054 km	43	7.61	5.65	5.83	39.63	<0.001
	Secondary cluster 3	2021/8/4 to 2021/8/10	18.684202 N, 99.158836 E / 0.74 km	58	14.45	4.01	4.18	37.93	<0.001
	Secondary cluster 4	2021/7/14 to 2021/7/20	18.694456 N, 99.165388 E / 0.18 km	69	23.62	2.92	3.05	29.54	<0.001
	Secondary cluster 5	2021/7/28 to 2021/8/3	18.697026 N, 99.165202 E / less than 0.1 km	20	2.25	8.89	9.03	26.09	<0.001
	Secondary cluster 6	2021/7/21 to 2021/7/27	18.699550 N, 99.164704 E / 0.17 km	45	14.36	3.13	3.22	21.18	<0.001
	Secondary cluster 7	2021/8/11 to 2021/8/17	18.689495 N, 99.148023 E / 0.097 km	35	9.73	3.6	3.68	19.82	<0.001
	Secondary cluster 8	2021/7/28 to 2021/8/3	18.703045 N, 99.162082 E / less than 0.1 km	20	3.37	5.93	6.02	19.09	<0.001

Secondary cluster 9	2021/7/28 to 2021/8/3	18.697799 N, 99.146628 E / 0.83 km	22	9.08	2.42	2.45	6.62	0.039
Secondary cluster 10	2021/7/28 to 2021/8/3	18.696043 N, 99.168316 E / 0.16 km	27	13.06	2.07	2.09	5.75	0.102
Secondary cluster 11	2021/7/28 to 2021/8/3	18.684388 N, 99.179936 E / 1.46 km	19	8.74	2.17	2.19	4.54	0.301
Secondary cluster 12	2021/8/4 to 2021/8/10	18.674529 N, 99.129612 E / 1.70 km	10	3.89	2.57	2.58	3.34	0.705

[1] MRCS=maximum reported cluster size; [2] O=observed case; [3] E=expected case; [4] O/E ratio=the ratio of observed cases/expected cases; [5] RR=relative risk; [6] LLR=log-likelihood ratio.
