# Supplementary material for: Identifying the patterns and sizes of the first lumpy skin disease outbreak clusters in Northern Thailand with a high degree of dairy farm aggregation using spatio-temporal models
Source: PLoS One. 2023 Nov 15;18(11):e0291692. doi: 10.1371/journal.pone.0291692 (PMC10651038; doi:10.1371/journal.pone.0291692)
Supplement: S3 Table — (PDF) [file pone.0291692.s003.pdf]

Supporting information 3

S3 Table. The most likely clusters detected by the Bernoulli scan statistic model of the first LSD outbreak in northern Thailand's dairy, 2021

MRCS[1]	Cluster type	Cluster time	Centroid(X,Y)/ Radius(km)	O[2]	E[3]	O/E ratio[4]	RR [5]	LLR[6]	p-value
**50%**	Most likely cluster	2021/7/28 to 2021/8/3	18.707028 N, 99.157001 E / 1.71 km	21	15.56	1.35	1.41	6.75	0.029
**25%**	Most likely cluster	2021/7/28 to 2021/8/3	18.699550 N, 99.164704 E / 0.55 km	14	10.37	1.35	1.39	4.39	0.287
	Secondary cluster 2	2021/7/28 to 2021/8/3	18.688837 N, 99.147418 E / 1.20 km	12	8.89	1.35	1.38	3.74	0.724
**10%**	Most likely cluster	2021/7/28 to 2021/8/3	18.702710 N, 99.165604 E / 0.47 km	8	5.93	1.35	1.37	2.46	0.96

[1] MRCS=maximum reported cluster size; [2] O=observed case; [3] E=expected case; [4] O/E ratio=the ratio of observed cases/expected cases; [5] RR=relative risk; [6] LLR=log-likelihood ratio.
